# Cognitive impairment and homelessness: A scoping review

**DOI:** 10.1111/hsc.12682

**Published:** 2018-11-13

**Authors:** Beth Stone, Sandra Dowling, Ailsa Cameron

**Affiliations:** ^1^ University of Bristol Bristol UK

**Keywords:** brain injury, cognitive impairment, developmental disabilities, homelessness, scoping review

## Abstract

This paper reports the findings of a scoping review designed to identify research which has explored the relationship between cognitive impairment and homelessness. A systematic search of databases for articles published between 2007 and 2017 was conducted using keywords relating to cognitive impairments and homelessness. Sources were expanded using manual searches of citations and grey literature. Forty studies represented in 45 papers were selected for review using predefined inclusion criteria. Sources were subject to quality appraisal and data were extracted in line with review questions. Prevalence studies were over‐represented in the review, while qualitative data were lacking. Aetiology of impairments was delineated by acquired and developmental causes. A variety of measures were employed by studies which were not validated in homeless populations. Studies did not give sufficient consideration to co‐occurring disorders and overlapping symptoms between aetiologies. Because of these factors, it was difficult to conclude that all studies had accurately measured what they set out to; however, the evidence suggested that cognitive impairment was disproportionately over‐represented in homeless populations. Cognitive impairment was found to be both a risk factor to and perpetuator of homelessness. Risk factors for homelessness were similar to those of the general population, though exaggerated by sequelae of certain cognitive impairments. The results of this review suggest that more attention needs to be paid to the underlying socioeconomic disadvantages, persons with cognitive impairments face which may lead to homelessness. Further research should prioritise the voice of homeless persons with cognitive impairments, to better understand both causes of homelessness and effective methods of rehabilitation.

**What is known about this topic**

- Research indicates that cognitive impairments are over‐represented in homeless populations.
- Research focuses on individual cognitive impairments such as brain injuries or learning disabilities.
- There is insufficient recognition and support for cognitive impairments in homeless populations.

**What this paper adds**

- The first systematic scoping review to look comparatively at research examining cognitive impairments with different aetiologies in the homeless.
- Findings from this review question the usefulness of further prevalence studies, which do not consider the overlap of co‐occurring disorders and do little to improve the lives of homeless people with cognitive impairments.
- Findings from this review suggest risk factors to, and perpetuators of, homelessness for people with cognitive impairments are exacerbated by inherent socioeconomic disadvantages.

## 1 INTRODUCTION

The UK law defines an individual as homeless if they have no accommodation, or if it is not reasonable for them to continue occupancy of their accommodation (NAO, 2017). Types of homelessness are wide ranging and include rough sleeping, sofa surfing, living in hostels or shelters, or in unsuitable forms of temporary accommodation (Bramley, 2017). Homelessness is on the rise in the United Kingdom. Official figures for 2017 show a 44% increase in annual homelessness application acceptances since 2009/10 (Fitzpatrick, Pawson, Bramley, Wilcox, & Watts, 2017), and a count of 4,134 rough sleepers in 2016 displays a 134% rise over the last 6 years (National Audit Office, 2017). It is of concern that these figures only reflect statutory homeless, which is quantifiable due to interaction with government agencies or specific services. In 2011, the charity Crisis reported that 63% of single homeless persons were “hidden” from support and statistics (Reeve, 2011).

The consequences of homelessness for both the individual and society are damaging. A health audit in 2014 found that 80% of homeless persons self‐reported a mental health issue and 41% long‐term physical health problems (Homeless Link, 2014). Malnourishment and substance (mis)use are also over‐represented in the homeless population (Homeless Link, 2014; Sprake, Russell, & Barker, 2013). From a societal perspective, factors associated with homelessness put a strain on resources. In 2010, the Department of Health reported that homeless hospital usage cost the NHS an estimated £85 million a year (Department of Health, 2010). Overall the cost of preventing homelessness is reported to be far lower than the cost of managing it (Pleace, 2015).

Homelessness is associated with a range of physical and mental health disparities including cognitive and neurological impairments (Backer & Howard, 2007; MacReady, 2009). For this review, cognitive impairment (CI) will be used as a broad term encompassing functional and clinical impairments which affect cognitive ability. Functional impairment refers to the affects a CI has on specific abilities such as problem‐solving, attention, or memory. Clinical conceptualisations concern diagnoses such as dementia, traumatic brain injury (TBI), learning or intellectual disability (learning disability), and autism spectrum disorder (ASD) (Centre for Persons with Disabilities, 2018). Such diagnoses are represented in the general population at moderate levels. The prevalence of learning disability in adults in England is conservatively estimated to be 2.16% (Public Health England, 2015). Dementia affects 1.3% of the entire UK population and 7.1% of those aged 65 and over (Prince et al., 2014).

Regardless of aetiology, many of the functional aspects of CIs and the way they are understood and supported by society result in poor social and economic outcomes. While not all people experience the same challenges, certain CIs are associated with communication, emotional, and adaptive functioning difficulties (Headway, 2018; NAS, 2018a). Furthermore, persons with CIs, as with other disabilities, are often misunderstood, undersupported, and subjected to prejudice at both individual and policy level (Aiden & McCarthy, 2014; EHRC, 2017). Such experiences can inhibit a person's ability to find employment, maintain relationships, apply for welfare, or understand how to pay rent or bills (Backer & Howard, 2007). In turn, this may prevent a person securing or maintaining suitable housing. Once homeless, it is difficult to identify and appropriately support individuals with CIs. Persons with CI can experience higher rates of mental health problems which are aggravated by homelessness (Eapen, 2014; Homeless Link, 2014), making it hard to distinguish underlying issues.

The limited research in this field tends to approach CI through the lens of brain deficit or diagnosis. Two known reviews have examined the prevalence of generalised CI in homeless populations (Burra, Stergiopoulos, & Rourke, 2009; Depp, Vella, Orff, & Twamley, 2015). It is hoped that by expanding such comparisons, we can better understand how to identify, support, and rehabilitate homeless people with CI. By employing systematic methodology, this review thus sought to (a) summarise existing research on CI and homelessness and identify gaps in knowledge, (b) ascertain how common CIs are in homeless populations and how they are measured, (c) identify risk factors to and perpetuators of homelessness for individuals with CIs. This article begins with a brief explanation of the epistemic framework to this study before setting out methods. Findings of this scoping review are followed by a discussion of context, including ramifications for current practice and policy.

### 1.1 Epistemological framework

The social model of disability distinguishes between impairment and disability, understanding the latter to be structured by social oppression, inequality, and exclusion (Thomas, 2004). Although the variations of the social model are commonly adopted in disability research, it is often posited as dichotomous to a biomedical understanding of disability and criticised for denying the importance of embodiment (Oliver, 2012). Alternatively, disability can be understood in a social‐relational way. This acknowledges that some restrictions of ability are down to impairment effects (Thomas, 2004), but that those which are “disabling” are caused by socially constructed barriers. This epistemic framework underpins this review and is returned to later in the article.

For the purpose of this review, the terminology “cognitive impairment” (CI) has been adopted. This phrasing reflects existing literature, although it is noted such a deficit‐focused language oversimplifies the aetiologies discussed.

## 2 METHODOLOGY

A scoping review is useful for determining the size and nature of a given knowledge base and can identify gaps in the literature for further research (CRD, 2008). While scoping review methodology was employed in a number of ways, for this study, a review was conducted in accordance with Social Care Institute for Excellence (SCIE) criteria (Rutter, Francis, Coren, & Fisher, 2013). This approach involves a detailed and systematic literature search to identify relevant papers followed by a process of data extraction and analysis.

### 2.1 Search strategy

A systematic search of The Cochrane Library, MedLine, PsycInfo, Scopus, Social Care Online and Web of Science was conducted for literature published in English between 2007 and 2017. To cover a wide range of cognitive impairment, “homeless*” was searched for in combination with the following terms: autis*, asperger*, ASD, brain injury, TBI, ABI, cognitive impairment, cognitive function*, neurodevelopmental, NDD, learning disab*, intellectual disab*, ADHD, attention deficit, down's syndrome, dementia, william*^1^. Searches were limited to abstract, title, and keywords and were modified where appropriate to yield more relevant or wider scoping results. While multiple terms were used to denote CI, pilot searches indicated that papers examining specific types of homelessness such as rough sleeping or staying in shelters included “homeless*” in their title, abstract, or keywords. Synonyms and types of homelessness were therefore removed to avoid erroneous results. Database searches yield a total of 625 sources. Manual searches were performed using reference lists derived from database results. This was accompanied by a search of grey literature and known authors through the knowledge of the field. Internal search tools of homeless charity, government, and media websites were used to locate potential material. This led to the inclusion of two research studies not published in academic journals (Campbell, 2015; Pritchard, 2010) and also provided supplementary sources which were used to corroborate findings.

Known reviews had included quantitative papers focussed on the prevalence of CI in homeless populations. Given the commitments to a social‐relational understanding of disability, and the aim to identify risk factors to homelessness for CI populations, it was decided that qualitative papers are included in the review.

### 2.2 Study selection

The search began on 1 November 2017 and concluded on 31 November 2017. Identified studies went through three stages of screening. Initially titles were scanned and subjected to the inclusion and exclusion criteria set out in Table 1; duplicates were removed at this stage. Exclusion and inclusion criteria were then applied via an abstract screening, after which texts were read in full. The rational for inclusion or exclusion of a source was founded upon the initial review questions and structural restraints of the review. For example, due to this review's focus on homeless adults, studies which exclusively sampled children or adolescents under the age of 18 were removed. Manual search results (*n* = 5) were added to database searches giving a combined total of 40 studies which were represented across 45 papers, this process is documented in Figure 1.

**Table 1:** Inclusion and exclusion criteria

#	Inclusion/Exclusion Criteria	Guidance
1	Include: homeless* in abstract or title AND (autis* or asperger* or ASD or brain injury or TBI or ABI or cognitive impairment or cognitive function* or neurodevelopmental or NDD or learning disab* or intellectual disab* or ADHD or attention deficit or down's syndrome or dementia or William*) in abstract or title.	Modify for databases as appropriate
2	Exclude: published before 2007	2007–2017 inclusive
3	Exclude: language not English	Seek translation where possible
4	Exclude: does not address review questions	Reviewer's judgement
5	Exclude: focus on children/youth	Exclusive <18 focus
6	Exclude: Specific intervention or program evaluation	Where non‐applicable across settings
7	Exclude: Irrelevant	Picked up by errant search
8	Exclude: Review or commentary of study	Find original where relevant
9	Exclude: Dissertation/thesis	Search for subsequent publication
10	Exclude: Not methodologically robust	Insufficient methodological detail, unable to apply quality appraisal

**Figure 1:**
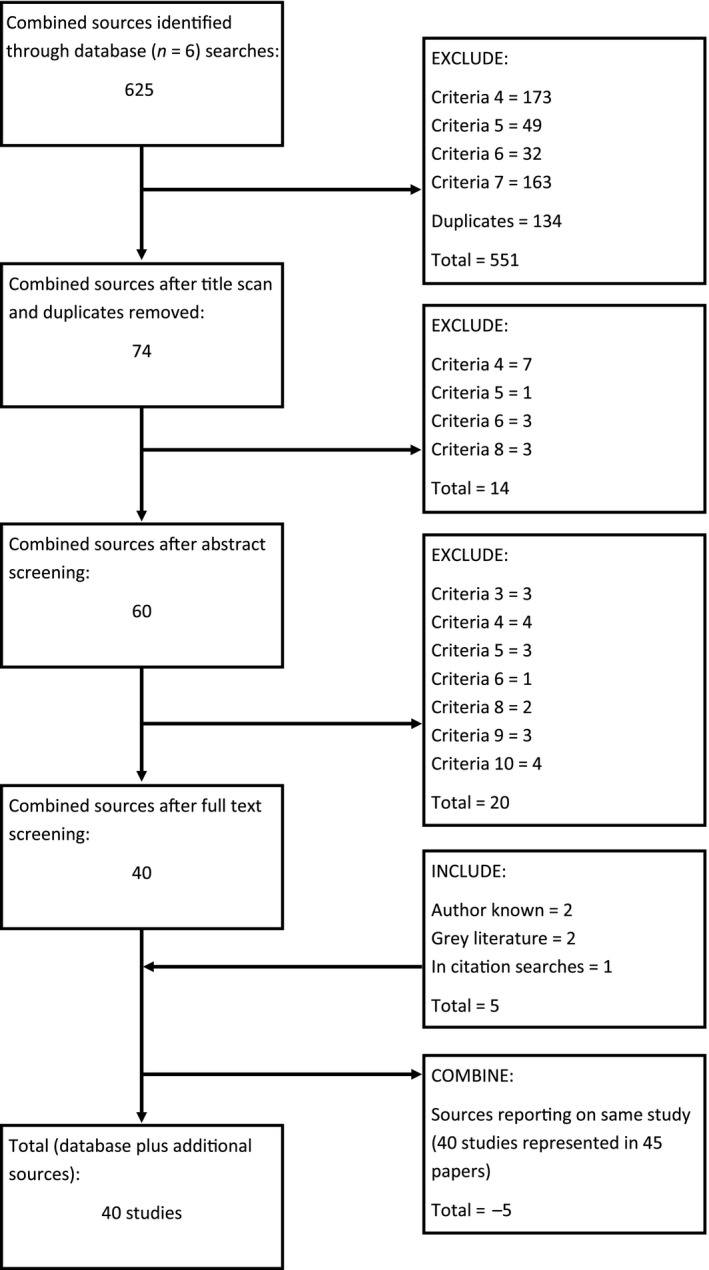
Literature flow chart

### 2.3 Quality appraisal

Included studies went through a process of quality appraisal and were given a rating of poor, fair, good, or very good. Due to methodological differences, studies were categorised by type and rated using distinct criteria. Criteria for appraisal was adapted where appropriate from multiple sources (Burra et al., 2009; CASP, 2017; CRD, 2008; Higgins & Green, 2008; Rutter et al., 2013). Qualitative papers, for example, were rated on research design, consideration of ethical issues, and centrality on user views, among other factors. Papers focussed on prevalence were assessed according to sample size, adjustment for confounding factors, and comparisons to matched control groups, among other factors. Studies were given a point for each matched criterion resulting in a score of up to 12 (systematic reviews) or 14 (prevalence, cohort, and qualitative studies).

All systematic reviews were rated good or very good. A greater range was displayed in empirical studies within which seven studies were poor, 13 fair, nine good, and eight very good (Keyser & Mathiesen, 2010, appraised against both qualitative and prevalence criteria). All qualitative studies presented details of how informed consent was obtained, though this was found to be lacking elsewhere. Other limitations included small sample sizes, not describing refusals, and not providing sufficient demographic detail. Studies tended to use convenience sampling and did not make efforts to reduce sampling bias.

Studies were assessed on the information available, and unreported information was treated as a negative score. It is acknowledged that reporting limitations may have negatively impacted the quality ratings of some studies. As criteria were adapted from multiple sources to increase their relevance to study type, it may be difficult to draw comparisons between the ratings of studies with different methodological processes. With these points in mind, quality ratings were used as a guide for inclusion in this review, as opposed to an objective measure. In the results section of this review, good and very good papers account for 63.7% of references, fair papers for 24.2%, and poor papers for 12.1%. Limitations of included papers have been noted where appropriate, with particular reference to those of poor quality.

## 3 RESULTS

### 3.1 Description of included studies

A total of 40 studies were included. These consisted of 36 empirical studies and four systematic reviews.

Thirty‐two studies were quantitative and primarily concerned with screening for CIs. Three qualitative and one mixed methods paper were also identified. Empirical studies mostly (*n* = 29) recruited from homeless populations, with definitions of homelessness ranging from absolute homelessness to recently rehoused, or in unsecure or unsuitable accommodation. Additional sample criterion included veteran status (Barnes et al., 2015; Brenner et al., 2017) and older age (Brown et al., 2017; Brown, Kiely, Bharel, & Mitchell, 2012; Hurstak et al., 2017; Joyce & Limbos, 2009; Okamura et al., 2017). Of studies which did not sample from homeless populations, two recruited from prisons (Hennessey, Stein, Rosengard, Rose, & Clarke, 2010; McCarthy et al., 2016), and other sampling frames included professionals who worked with the homeless (Colman, Hebblethwaite, Hames, Forsyth, & Donkin, 2007), mental health service clients (Keyser & Mathiesen, 2010), and records of hospital admissions (McMillan et al., 2015). These studies were included because they directly reported on participants with CIs experiencing homelessness. Sample size ranged from 12 to 1,500 participants, with larger samples tending to be recruited from ongoing cohort or RCT studies of the homeless (Nikoo et al., 2017; Stergiopoulos et al., 2015; Van Straaten et al., 2017, 2014 ). Some papers reported on the same project or sample. These papers are at times reported on independently due to differences in sample type (Colman et al., 2007; Hebblethwaite, Colman, Hames, Forsyth, & Donkin, 2007) and measures or methodology employed (Barnes et al., 2015; Brenner et al., 2017; Brown et al., 2017; Hurstak et al., 2017; Pluck et al., 2011, 2012 ; Van Straaten et al., 2017, 2014 ).

Four systematic reviews were included. Studies reviewed included those reporting on cognitive dysfunction tests (Burra et al., 2009), measures of cognitive ability (Depp et al., 2015), memory impairment (Ennis, Roy, & Topolovec‐Vranic, 2015), and TBI (Topolovec‐Vranic et al., 2012) in homeless populations. All reviews noted the scarcity of published empirical research which met their inclusion criteria; number of included studies ranged from 22 to 24 for general cognitive deficit, 11 for memory impairment, and eight for TBI.

The process of data extraction identified three main focuses concerning presence or cause of CI. As such, studies have been grouped according to the CI or aetiology they examined. Category A, summarised in Table 2, contained studies concerned with the presence of global cognitive deficits, or specific brain deficits such as difficulties with executive functioning or frontal lobe impairments. Category A studies did not directly investigate the cause of such deficits. Category B, summarised in Table 3, included studies concerned with acquired impairments. This category covered CIs which developed post childhood or were caused by nonorganic factors. The studies in Category C, summarised in Table 4, focused on CIs with developmental origin, these included attention deficit hyperactivity disorder (ADHD), learning disability, and ASD.

**Table 2:** Category A: General and unspecified cognitive impairment

Reference	Sample size	Sample type	Country	Research design	Main cognitive measures (abbreviations in table 2.4)	Prevalence	Quality rating
Burra, et al. (2009)	n/a	Review of 22 studies focussed on adults aged over 18, recruited from range of homeless (HL) services	International	Systematic review	MMSE score—range from 22 studies	4%–7% CI	Good
Depp, et al. (2015)	2,969 (pooled)	Review of 24 studies, mean sample age of 46.1, recruited from range of HL services	International	Systematic review	Cognitive impairment rates pooled from 24 studies	25% CI	Good
Ennis, et al. (2015)	n/a	Review of 11 studies, mean sample age of 40.75, recruited from range of HL services	International	Systematic review	n/a	*n*/a	Good
Joyce and Limbos (2009)	49	HL adult males aged 55 or older living in a HL shelter	Canada (Toronto)	Cross‐sectional study, interviews Review of medical records	MMSE	37.9% CI	Poor
Nishio, et al. (2017)	114	HL adults with mean age of 54, recruited with help of support centre.	Japan (n/a)	Diagnostic interview Questionnaire	WAIS III	34.2% CI	Fair
Okamura, et al. (2017)	51	HL adult males aged 65 or over living in HL shelters	Japan (Tokyo)	Psychiatric Interviews	MMSE	78.4% CI	Poor
Rogoz and Burke (2016)	171	HL adults aged 45 and over recruited from shelters, hospitals, hostels and housing agencies	Australia (Sydney)	Cognitive impairment screening Diagnostic interview	MMSE Battery of CI tests—clock drawing test, verbal fluency test, TMT	78% CI	Fair

**Table 3:** Category B: Acquired cognitive impairments

Reference	Sample size	Sample type	Country	Research design	Main cognitive measures (abbreviations in table 2.4)	Prevalence	Quality rating
Andersen, et al. (2014)	34	Homeless (HL) adult males with mean age of 58.8, recruited from urban shelter	Canada (Toronto)	TBI screening, TBI/no TBI comparison	BISQ RBANS	35% TBI	Fair
Barnes, et al. (2015) Brenner, et al. (2017)	229 309	Veterans with mean age of 51.8 (2015) and 52.3 (2017) seeking HL services at metropolitan health care systems	USA	TBI screening, Interview Psychiatric interviews, TBI/no TBI comparison	OSU TBI‐ID OSU TBI‐ID	90.4% TBI 91% TBI	Good
Brown, et al. (2017) Hurstak, et al. (2017)	350 343	Older HL adults with mean age of 58. Recruited from overnight shelters, homeless encampments and recycling centres	USA (Oakland, CA)	Interviews and condition assessments Structured interviews and testing	MMSE 3MS TMTB	25.8% CI 25.1% CI	Very good
Brown, et al. (2012)	247	HL adults aged between 50 and 69 recruited from 8 Hl shelters	USA (Boston, MA)	Assessment interviews, compared to 3 cohorts	MMSE TMT‐B	24.3% CI	Good
Bymaster, et al. (2017)	127	HL adults with mean age of 48, accessing healthcare at two clinic sites.	USA (San Jose, CA)	Questionnaire	OSU TBI‐ID	77% TBI 58% TBI with loss of consciousness	Fair
Gargaro, et al. (2016)	48	Clients of service for HL people with mental health problems and/or substance abuse issues aged 25 to 71 (mean of 43.40)	Canada	TBI Screening	OSU TBI‐ID	56% TBI	Poor
Hwang, et al. (2008)	904	HL adults with mean age of 37.4, surveyed at shelters and meal programs	Canada (Toronto)	Cross sectional survey, face to face interview, TBI/no TBI comparison	TBI screening adapted from prison inmate survey	53% TBI 23% moderate or severe TBI	Very good
McMillan, et al. (2015)	1590 records	Records of HL registered at NHS General Practitioner health services (in proximity of HL services)	United Kingdom (Glasgow)	Data comparison of hospital admissions for head injury and death records	*n*/a	13.5% Hospitalised with head injury	Good
Nikoo, et al. (2017)	1,190	HL and vulnerably housed adults aged 18 or over. Recruited from Health and Housing in Transition study	Canada	Assessment followed by yearly structured interviews for 3 years	TBI screening adapted from prison inmate survey	60.8% TBI	Very good
Oddy, et al. (2012)	200	HL adults with mean age of 32.73, recruited from homeless hostels and day centres	United Kingdom (Leeds)	Cross‐sectional survey design, questionnaire	TBI screening adapted from prison inmate survey	48% TBI	Good
Pluck, et al. (2011) Pluck, et al. (2012)	55 80	HL adults recruited from HL services including shelters, day centres and meal services	United Kingdom (Sheffield)	Interviews and assessments Diagnostic interview	FrSBe WASI WTAR WMS WASI	75% neurobehavioural impairment IQ: 84.3 19.2% in extremely low range	Fair
Pluck, Nakakarumai, and Sato (2015)	16	HL adult males with mean age of 51.5 recruited from singular homeless service (day centre and outreach program)	Japan (Tokyo)	Cognitive examination—premorbid IQ, Frontal lobe function, and global CI	JART WCST MMSE	44% CI	Fair
Svoboda and Ramsay (2014)	170	Homeless and low‐income men with mean age of 43.7, recruited from hostels and low‐income housing sites	Canada (Toronto)	Collection of emergency department records of head injury, interviews	DSM mental health criteria	*n*/a	Very good
To, et al. (2015)	1,181	HL and vulnerably housed adults with mean age of 43.0 recruited from shelters and meal programs across 3 cites	Canada (national)	Structured interviews at baseline and 1 year after	Hwang et al. (2008) TBI questionnaire	61% TBI	Very good
Topolovec‐Vranic, et al. (2012)	*n*/a	Review of 8 studies, looking at HL and TBI. Included studies which recruited from range of HL and health services, mean sample age of 36.2	International	Systematic review	*n*/a	8%–53% TBI	Very good
Topolovec‐Vranic, et al. (2014)	111	Males with mean age of 54.2 recruited from singular large urban HL shelter	Canada (Toronto)	Semi‐structured interview, TBI/no TBI comparison	BISQ	45% TBI	Very good
Topolovec‐Vranic, et al. (2013)	37	HL stakeholders including frontline workers, healthcare professionals, policy makers, students and person's previously HL	Canada (Toronto)	Multidisciplinary TBI stakeholder workshop	*n*/a	*n*/a	Fair
Stergiopoulos, et al. (2015)	1,500	HL adults with mean age of 41.1. Recruited from “At Home” study, where participants were referred from range of HL services	Canada (national)	Neuropsychological assessment	Digit Symbol Coding from WAIS‐R TMT (parts A and B) HVLT‐R	72% CI	Good

**Table 4:** Category C: Developmental cognitive impairment

Reference	Sample size	Sample type	Country	Research design	Main cognitive measures (abbreviations in table 2.4)	Prevalence	Quality rating
Campbell (2015)	12	Adults who had previously experienced homelessness (HL)	United Kingdom (Wales)	Literature and policy review, qualitative interviews	*n*/a	*n*/a	Fair
Colman, et al. (2007) Hebblethwaite, et al. (2007)	23 14	Professionals who worked with HL\HL with learning disability known to social or health services	United Kingdom (England)	Qualitative interviews	*n*/a	*n*/a	Good
Evans (2015)	415	Individuals with autism and their families	United Kingdom (Wales)	Online survey	*n*/a	12% of ASD adults experienced homelessness (EHL)	Poor
Garcia Murillo, Ramas‐Olazagasti, Mannuzza, and F. X., Klein, R.J. (2016)	207 (134 ADHD)	Clinically referred male children with ADHD, mean age 8, followed up at mean age 41	USA	Longitudinal follow‐up	DSM criteria for ADHD	23.9% of ADHD EHL	Good
Gouveia, et al. (2017)	71	HL adults with mean age of 37.83	Mozambique (Maputo, Matola)	Psychiatric assessment and treatment	Hospital assessment, MINI	5.6% ID	Poor
Hennessey, et al. (2010)	192	Incarcerated women with mean age of 33	USA (Rhode Island)	Questionnaire, face‐to‐face interviews	WURS	40% of ADHD EHL in year prior	Very good
Keyser and Mathiesen (2010)	37	Clients from two social service agencies providing outpatient treatment, interview participants had a mean age of 43.9	USA	Background questionnaire, Semi‐structured interviews with learning disability participants (*n* = 9)	LNST	100% EHL 33% HL over 1.5 months.	Poor (prevalence) Fair(qualitative)
McCarthy, et al. (2016)	240	Male prison inmates NDD prisoners had a mean age of 31.3	United Kingdom (England)	Face‐to‐face interviews, assessment for NDDs	LDSQ, 20‐item autism quotient, adult ADHD self‐report scale, self‐reported diagnosis	36% NDD 23% HL before imprisonment	Fair
Mercier and Picard (2011)	68	Adults with learning disability referred by HL service provider, mean age 43	Canada	Characteristics and demographics of learning disability compared with general homeless population	*n*/a	*n*/a	Good
Nishio, et al. (2015)	18	HL adults with mean age of 56.8, recruited from meal service and social welfare centre	Japan (Nagoya)	Psychiatric interviews	WAIS III, MINI, JART, Adult ADHD self‐report scale	39% learning disability 17% possible NDD	Fair
Oakes and Davies (2008)	50	HL adults with mean age of 33.6, recruited from a general practice providing HL services	United Kingdom (England)	Assessment interview and file review	WASI, ABAS	6/50 (12%) learning disability	Fair
Patterson, et al. (2012)	497	Absolutely homeless or vulnerably housed with mental health problems , mean age 40.8	Canada (Vancouver)	Questionnaire	Self‐reported learning disability in childhood	27% learning disability	Good
Pritchard (2010)	14	Entrenched rough sleeper clients taking part in rehousing program	United Kingdom (England)	Evaluation of individualised budget program, in‐depth interviews, and structured questionnaire	*n*/a	50% ASD	Fair
Tripathi, et al. (2013)	140	HL adults with mean age of 34.6, recruited from the streets	India	Assessment of clinical profiles of mentally ill homeless inpatients, treatment, and rehabilitation	Psychiatric disorders—ICD−10 Intelligence assessments—unspecified	39% learning disability	Poor
Van Straaten, et al. (2014) Van Straaten, et al. (2017)	387 513 *t*2 = 336	HL adults with mean ages of 39.9 (learning disability 2014) and 40.7 (learning disability 2017). Recruited from social relief services and temporary accommodation	Netherlands	ID prevalence and substance (mis)use screening Participants interviewed at baseline and 1.5 years later. Screened for ID and interviewed regarding care needs	HASI HASI	29.5% suspected learning disability 31% at t2 had suspected learning disability	Very good

### 3.2 Measure and prevalence of cognitive impairment

This section describes the types of CIs examined by included studies. Prevalence rates are presented where available along with the measures or screening tools utilised. A table summarising the measures employed by prevalence studies is found in Table 5.

**Table 5:** Cognitive measures

Abbreviation	Elaboration	Description of measure
—	ADHD self‐report scale	Self‐report checklist devised from DSM criteria
ABAS	Adaptive Behaviour Assessment Scale	Measure of adaptive behaviour of everyday life function across 10 domains
BISQ	Brain Injury Screening Questionnaire	Three‐part structured questionnaire. Reliant on self‐recall. Widely and clinically used
DSM	Diagnostic and Statistical Manual of Mental Disorders	Criteria for classification of mental disorders. Standardised definitions adopted clinically
FrSBe	Frontal Systems Behaviour Scale	46‐item rating scale to determine behavioural problems caused by frontal lobe damage
HASI	Hays Ability Screening Index	Short screening test for the presence of possible learning disability
HVLT‐R	Hopkins Verbal Learning Test‐Revised	Measure of verbal learning and memory using six forms to allow frequent retesting
ICD	International Classification of Diseases	International diagnostic tool for diseases. Includes criteria for mental and behavioural disorders
JART	Japanese Adult Reading Test	Reading test of Japanese characters. Estimates premorbid intelligence quotients
LDSQ	Learning Disability Screening Questionnaire	Short questionnaire to ascertain the presence of learning disability in adults
LNST	Learning Needs Screening Tool	Short screening tool designed to ascertain need for referral for further assessment/diagnosis
MINI	Mini International Neuropsychiatric Interview	Brief, structured diagnostic interview to ascertain the presence of psychiatric disorders
MMSE	Mini‐Mental State Examination	30‐point questionnaire to measure general cognitive impairment. Commonly used
OSU TBI‐ID	Ohio State University TBI Identification Method	Short structured interview to elicit history of TBI. Reliant on self‐recall. Widely and clinically used
RBANS	Repeatable Battery for the Assessment of Neuropsychological Status	Neuropsychological assessment for adults made up of 10 tests over five domains
TMT (A/B)	Trail Making Test (Part A/Part B)	Neuropsychological visual test where participants connect letters/letters and numbers in sequence
WAIS (III) (‐R)	Wechsler Adult Intelligence Scale (3rd Edition) (Revised)	Adult IQ test. Tests intelligence across four domains: verbal comprehension, perceptual organisation, working memory, processing speed
WASI	Wechsler Abbreviated Scale of Intelligence	Revision of WAIS. Gives brief assessment of cognitive ability in clinical, educational, and research settings
WCST	Wisconsin Card Sorting Test	Card sorting test considered a measure of executive function
WMS	Wechsler Memory Scale	Test to measure memory ability in adults
WTAR	Wechsler Test of Adult Reading	Reading test used to assess premorbid intelligence
WURS	Wender Utah Rating Scale	Assessment for ADHD in adults. 61 questions used to recall childhood behaviour (25 associated with ADHD)
3MS	Modified Mini‐Mental State Test	Extension of MMSE. Commonly used to discern severity of dementia
‐	20‐item autism quotient	Shortened questionnaire to ascertain the presence of autistic symptoms in adults of average intelligence

#### 3.2.1 Facets of cognitive disfunction

Studies in Category A measured facets of cognitive dysfunction. Although all studies had higher rates of CI in their samples in comparison to the general population, CI was broadly defined and screened for using a variety of measures. The most common measure used was the MMSE to assess for global cognitive deficits. Studies which employed the MMSE recorded impairment levels between 25% and 78%. Although the MMSE is often used to denote possible dementia, studies which employed the MMSE did not adopt further diagnostic measures. Memory deficits were found to be common in Ennis et al.’s (2015) review; however, no included studies adequately represented older people. Furthermore, and despite the suspected presence of dementia in their sample, Okamura et al. (2017) reported no difference in mean CI rate between participants below and above the age of 75.

Rogoz and Burke's (2016) study measured characteristics of CI using a battery of different tests. These, more specified measurements, still identified high rates of CI across their sample and significant impairment of frontal lobe function. Finally, using the WAIS, Nishio et al. (2017) found that 34.2% of their homeless sample was affected by CI although they did not examine aetiology.

#### 3.2.2 Traumatic brain injury

The most prominent of aetiologies in Category B was TBI. As Table 3 illustrates, 13 of 18 studies reported on brain injury in the homeless population. The OSU TBI‐ID and BISQ were used to ascertain prevalence of TBI as well as self‐report measures adapted from previous research. The prevalence of TBI ranged from 35% to 91% though such range may be partly accounted for by the scope of TBI definitions. Lowest rates of head injury were found by McMillan et al. (2015), who recorded hospital admissions for head injury in the homeless at 13.5%. This narrower definition of head injury, however, still gave a prevalence rate 5.4 times higher than the general population it was compared to. The highest rates of around 90% were found in the sample of veterans examined by Barnes et al. (2015) and Brenner et al. (2017) and may be partially accounted for by increased risk of TBI associated with serving in the armed forces.

Few studies investigated how head injury occurred; those which found assault to be the most common cause (Barnes et al., 2015; Gargaro, Gerber, & Nir, 2016; Topolovec‐Vranic et al., 2014). Cause of injury which was further examined by Topolovec‐Vranic et al. (2014) who found TBI in participants below 40 was mostly caused by substance misuse leading to a fall and those 40 and over by assault. TBI studies found the majority (70%–90%) of TBIs occurred prior to the onset of homelessness (Barnes et al., 2015; Hwang et al., 2008; Oddy, Moir, Fortescue, & Chadwick, 2012; Topolovec‐Vranic et al., 2014), though it was acknowledged that once homeless risk of TBI increased. Nikoo et al. (2017) found that over 3 years, 37% of their homeless cohort suffered a further TBI. Multiple head injuries were also found to be significantly higher in homeless populations by McMillan et al. (2015) and Oddy at al. (2012). Conversely, Svoboda and Ramsey (2014) who investigated the rates of head injury between the general homeless, chronically homeless with drinking problems (HCDP), and low income housed found having a head injury was better predicted by previous head injury, drug dependence, or a seizure disorder than a history of homelessness. They note, however, that among the HCDP group, rates of head injury were 400 times higher than in the general population, and that the time interval between injuries decreased with each successive head injury.

Although TBI is associated with CI, this was not routinely measured. Andersen et al. (2014) found association between TBI and poorer cognitive performance in their homeless sample. Other studies which noted high rates of moderate to severe head injuries (Barnes et al., 2015; Brenner et al., 2017; Hwang et al., 2008; Svoboda & Ramsay, 2014) suggested these related to ongoing neurological deficits.

#### 3.2.3 Other acquired causes

Acquired CIs resulting from “geriatric” conditions were measured in two studies by Brown et al. (2012), Brown et al. (2017). Both studies found around a quarter of their older sample had a CI, suggesting CI could develop due to age‐related syndromes. CI was also examined in relation to childhood trauma. Pluck et al. (2011) used the FrSBe and WASI to measure neurobehavioural impairment in a homeless sample, finding three quarters were in the clinically significant range. Low IQ and neurobehavioural deficit were found to be strongly associated with childhood trauma, with 89% of the sample reporting childhood abuse. A smaller sample from this study was also measured for CI. In their paper published in 2012, Pluck et al. explain how they used IQ as a measurement of cognitive functioning finding 19% of their sample was in the extremely low range in comparison to a 2.2% estimate in the general population. To further investigate homelessness and CI, in 2015, Pluck et al. used a battery of tests to ascertain a 44% global CI rate in a sample of 16 homeless adults in Japan. In both the 2012 and 2015 paper, it was suggested that impairments were acquired as opposed to developmental, due to higher estimates of cognitive functioning prior to participants becoming homeless. Substance abuse and mental health problems were noted as possible causes of this decline. In the 2012 study, however, half of participants’ prehomeless reading scores were so low that they could not be validly estimated, and 33.8% of participants had special educational needs as a child.

Two papers directly investigated the link between mental health problems or substance abuse and CI. Using a subset of Brown et al.’s, 2017 sample of older homeless adults, Hurstak et al. (2017) found high‐severity alcohol use was strongly associated with cognitive impairment. Furthermore, in a sample of 1,500 homeless adults with mental health problems, Stergiopoulos et al. (2015) recorded 72% CI, noting around half of their sample met criteria for psychosis, major depressive disorder, and alcohol or substance use disorder. Despite these correlations, both studies note that their cross‐sectional design limits implications of causality (Hurstak et al., 2017; Stergiopoulos et al., 2015). Stergiopolous et al. (2015) also acknowledge that much of the variation in their sample was unaccounted for, and other variables of interest, such as the presence of learning disability, were not tested for.

#### 3.2.4 Neurodevelopmental disabilities

Category C studies were concerned with developmental disability. Developmentally caused impairments included learning disability, ASD, and ADHD. Qualitative research was most prominent in this category.

Ten studies concentrated on the prevalence and support of learning disability in the homeless population. These studies used variations of standardised measures to assess prevalence. Core intellectual ability was measured using the WASI alongside adaptive functioning by Oakes and Davies (2008), and Wechsler‐based measures (WAIS III) were also adopted by Nishio et al. (2015). Other measures of learning disability included the LNST (Keyser & Mathiesen, 2010) and the HASI (Van Straaten et al., 2017, 2014). Prevalence rates of learning disability in the homeless ranged from 5.6% to 39%. Rates were compared with UK general population estimates of 2.5%–3% (Oakes & Davies, 2008) and Dutch estimates of 0.7% (Van Straaten et al., 2014). Although adults were not assessed for learning disability in Patterson's, Moniruzzaman, Frankish, and Somers (2012) study, 36% of participants reported an learning disability in childhood, a diagnostic criterion for learning disability. A significantly high percentage of 39% learning disability was also found in Tripathi et al.’s (2013) homeless sample; however, measures of assessment were not well documented. Studies which recruited learning disability participants supported these findings, with high rates of homelessness reported. In Keyser & Mathiesen's, 2010 study, all learning disability participants reported housing instability, with three of nine interview participants experiencing periods of homelessness averaging over 1.5 months. Reports of learning disability were strengthened in studies which collected life history or attempted to identify if learning disability had been present in childhood (Mercier & Picard, 2011; Oakes & Davies, 2008). The suggestion prevalence of CI in the homeless population may be partially accounted for by learning disability was also supported by one review (Depp et al., 2015), while the authors did not attempt to ascertain cause of CI, they note a below average mean score in studies that estimated premorbid IQ.

Other developmental CIs examined were ASD and ADHD. Using standardised tools (LDSQ, ADHD self‐report scale, 20‐item Autism Quotient), McCarthy et al. (2016) screened a sample of male prisoners for learning disability, ASD, and ADHD. They found 36% of their sample had at least one neurodevelopmental disability (NDD), and 23% of these individuals had experienced homelessness before imprisonment. Studies also showed that persons with ADHD reported high levels of homelessness. In 2016, Garcia Murillo et al. reported on a 33‐year follow‐up study, noting that 23.9% of their ADHD sample had experienced homelessness. This was further supported by Hennessey et al. (2010), who in their study of ADHD prevalence in incarcerated women found 40% of those with ADHD had been homeless in the year prior to incarceration. Using discrepancies on the WAIS III scores, Nishio et al. (2015) found 16% of their small homeless sample had suspected ASD, though no life history was available to aid diagnosis. Although no other study directly measured ASD in a homeless population, two studies found a disproportionate amount of autistic people experience homelessness. In 2010, when evaluating a rehousing program in the United Kingdom, Pritchard notes 7 of 14 rough sleepers registered on the autistic spectrum. Although diagnostic detail is lacking, it appears that participants were diagnosed through a mixture of the author's clinical expertise and previous diagnosis. Furthermore, in a large survey of autistic adults and their families in Wales, 12% of autistic respondents self‐reported experiencing homelessness since leaving school (Evans, 2015).

### 3.3 Risk factors and perpetuators

Risk factors and perpetuators of homelessness were often overlooked by studies where emphasis was placed on measuring prevalence. Those which were identified are examined below.

Economic success was negatively associated with all types of CI. The deficits following TBI such as with memory, attention, or planning made it difficult to maintain employment and housing (Anderson et al., 2014; Oddy et al., 2012). Low employment rates following a TBI were suggested as exacerbating circumstances and leading to homelessness (Topolovec‐Vranic et al., 2013). Similarly individuals with developmental disabilities had high levels of job instability (Hennessey et al., 2010; Keyser & Mathiesen, 2010; McCarthy et al., 2016). Low levels of educational attainment and inconsistent employment were also suggested as leading to poverty and subsequent homelessness (Hennessey et al., 2010). Many prevalence studies found that within homeless populations levels of educational attainment were lower for those with CI (Hurstak et al., 2017; Patterson et al., 2012; Rogoz & Burke, 2016; Stergiopoulos et al., 2015; Van Straaten et al., 2017). CI was also linked to contact with the Criminal Justice System. History of TBI was found to be independently and significantly associated with arrest and incarceration (To et al.., 2015; Topolovec‐Vranic et al., 2014) and persons with NDDs were over‐represented in the prison population (Hennessey et al., 2010; McCarthy et al., 2016).

Relationship breakdown was commonly cited as a cause of homelessness. Breakdowns could be the result of the death of caregiver, commonly a parent, with no appropriate support system in place, or as a result of social deficits such as difficulties building and maintaining relationships or vulnerability to abuse (Campbell, 2015; Hebblethwaite et al., 2007). Relationship breakdown was mostly associated with developmental impairments (Campbell, 2015; Gouveia et al., 2017; Mercier & Picard, 2011), though one study suggested emotional and family problems resulting from TBI as a cause of homelessness (Gargaro et al., 2016). Relationship difficulties were also considered to perpetuate homelessness by both individuals with learning disabilities and service providers working in the homelessness sector (Colman et al., 2007; Hebblethwaite et al., 2007). Lastly, cultural differences were considered significant when a learning disability was seen as “incurable” or a “burden,” as homeless individuals were less likely to be reintegrated with their families (Gouveia et al., 2017).

Studies reported high rates of mental health problems (Brenner et al., 2017; Brown et al., 2017, 2012 ; Campbell, 2015; Depp et al., 2015; Gargaro et al., 2016; Gouveia et al., 2017; Hebblethwaite et al., 2007; Hwang et al., 2008; Joyce & Limbos, 2009; Mercier & Picard, 2011; Nikoo et al., 2017; Nishio et al, 2017, 2015; Okamura, 2017; Patterson et al., 2012; Pluck et al., 2012; Rogoz & Burke, 2016; Topolovec‐Vranic et al., 2012, 2014 ; Tripathi et al., 2013; Van Straaten et al., 2014). Substance (mis)use was also common across samples, though variation of definitions and methods employed meant aggregate figures could not be provided. Persons with a TBI were more likely to be using multiple substances (Gargaro et al., 2016) or misuse drugs and/or alcohol (Hwang et al., 2008; Nikoo et al., 2017; Svoboda & Ramsey, 2014; Topolovec‐Vranic et al., 2014). Substance dependence was also associated with those with developmental CIs (Mercier & Picard, 2011; Van Straaten et al., 2014). In Mercier and Picard's (2011) study, a third of their learning disability sample attributed homelessness to substance abuse. The complex interplay between mental health, substance (mis)use, CI, and homelessness was not directly explored in papers, but acknowledged, and seen as exacerbating existing problems (Mercier & Picard, 2011; Pluck, 2011). Overall co‐occurring conditions were not seen to be adequately supported. Campbell (2015) noted that though eight of her 12 autistic participants had a co‐occurring condition, only one was receiving support for this at the time of homelessness. Tripathi et al. (2013) also highlighted untreated mental health problems as the main cause and perpetuator of homelessness.

Some sequalae associated with CIs were seen as prolonging homelessness or as barriers to rehabilitation. Pritchard (2010) explained how his autistic clients had grown used to the routine of living on the streets, making it difficult for service providers to successfully rehabilitate. learning disability needs were found to be similar to needs of non‐learning disability homeless, but were enduring as opposed to temporary (Van Straaten et al., 2017). Furthermore, it was suggested persons with TBI may struggle to interact appropriately with services and find it difficult to commit to program rules (Brenner et al., 2017). These examples suggested that housing difficulties resulted from impairment‐associated behaviours or symptoms.

Barriers to rehabilitation were not always internalised, as some studies highlighted the difficulties homeless services had meeting the needs of individuals with CIs. These studies reported a lack of awareness of CIs, a lack of specialised programs to treat CI and co‐occurring conditions, and inappropriate and low‐quality housing which was unsustainable in the long term (Colman et al., 2007; Hebblethwaite et al., 2007; Oakes & Davis, 2008; Topolovec‐Vranic et al., 2013). Difficulties in accessing support were also highlighted for persons with mild or “high‐functioning” ASD who were either undiagnosed, experiencing long delays during the diagnostic process, or did not meet the threshold for care or mental health services (Campbell, 2015; Pritchard, 2010). These findings reflect a social‐relational understanding of CIs, in that risk factors to homelessness do not solely arise from individual pathology or symptoms but also from the way such individuals are supported.

## 4 DISCUSSION

The following discussion is split into three sections. Initially, the identification and prevalence of CI in homeless populations is considered alongside current debates on the classification and recognition of specific aetiologies. Second, experiences of homelessness are discussed, examining how risk factors to homelessness for persons with CI compare with the general population. This discussion concludes with a consideration of implications for future research, service delivery, and policy.

### 4.1 Identifying cognitive impairment

#### 4.1.1 Prevalence

Although the variety was displayed in the methods used and outcomes measured by included studies, CI was found to be over‐represented in the homeless population. Similarly, studies which sampled specific types of CI noted higher rates of experiences of homelessness than in the general population. In the United Kingdom, these results substantiate anecdotal reports in the media and charity publications which allude to growing numbers of individuals with CI experiencing homelessness (Homeless Link, 2017; Innovation & Good Practice Team, 2015; Trueland, 2009).

#### 4.1.2 Accuracy of measures

Most studies claimed to use “standardised” or “widely accepted” measures. Although some measures had been designed for use with vulnerable groups (HASI, van Straaten et al., 2014), or validated in brain‐disordered populations (HVLT‐R, Stergiopolous et al., 2015), none had not been adequately validated in homeless populations. The MMSE was commonly used to ascertain CI but is too general to identify focal cognitive impairments (Backer & Howard, 2007; Burra et al., 2009). Studies which solely employed the MMSE had little justification for discussion of aetiology and CI affects based on such generalised prevalence rates. Self‐report mechanisms were also widely adopted, particularly for studies determining TBI. Although limited capacity should not be assumed in populations with CI, it is acknowledged that the presence of CI or circumstances surrounding homelessness such as substance abuse could affect an individual's ability to accurately self‐report. None of the included papers explored this possibility satisfactorily.

In determining the presence of learning disability or ASD, access to medical records or life history is especially useful. This can be difficult when sampling older, homeless populations who may not possess or be able to retrieve such information. In lieu of this, some studies relied upon the author's clinical expertise (Pritchard, 2010) or overly inclusive screening tools (Nishio et al., 2015; Van Straaten et al., 2014). It is possible that such studies overestimated the levels of developmental disability in their samples. However, a history of reduced contact with services may also decrease the likelihood of developmental disabilities being picked up earlier on in life. Campbell (2015) noted in her study of autistic adults only 58% had an official diagnosis at the time of homelessness. This may be partially accounted for by the fact that autism screeners have not been sufficiently adapted for diagnosis in adults (Mukaetova Ladinska, Perry, Baron, & Povey, 2012), and that "higher functioning" adults with learning disabilities and autism have been shown to develop coping mechanisms which mask their disabilities. Given these considerations, it is also possible that learning disability and ASD were under‐represented in some studies.

#### 4.1.3 Diagnostic boundaries and overlapping symptoms

Studies used differing definitions of CI. Some differences were internal, where adoption of screening measures necessitated a particular definition of an aetiology. This was seen most clearly in TBI studies where loss of consciousness or hospitalisation could determine the classification of a head injury but was not consistent between studies. Differences in definition were also contextual and related to temporal understandings of the impairment in question. The updating of the DSM‐IV to DSM‐V, for example, included diagnostic changes which may limit the generalisability of included studies. Furthermore, it is likely that conceptualisations of cognitive impairments may differ according to culture. Although it is not within the scope of this review to debate the accuracy of diagnostic criteria, the context of individual studies must be considered when comparing international research over a 10‐year period.

A further issue with studies which determined the presence of specific CIs is the recognition that aetiologies are not mutually exclusive. This is exemplified in Bymaster's , Chung, Banke, Choi, and Laird (2017) TBI study in which demographic tables describe high rates of diagnosed learning disability or special education in childhood. It is widely accepted that CIs often occur concurrently. ASD, for example, may be diagnosed alongside learning disability, Down's Syndrome, and ADHD (NAS, 2018b). Furthermore, some of the aetiologies considered in this review share similar symptoms and diagnostic criteria. The effects of TBI on executive functioning, in particular the ability to plan and organise, resonate with known ASD symptoms (Singh et al., 2016). Frontal lobe impairment, as examined by Rogoz and Burke (2016), is associated with learning disability, TBI, and dementia. Despite these considerations, studies tended to examine aetiologies exclusively.

Homelessness is associated with mental health problems and substance use disorders, issues commonly experienced alongside CIs. Although it is thus unsurprising that studies reported very high rates of mental health and substance misuse disorders, it is possible that the interplay between these factors further complicates the identification and classification of specific CIs. Backer and Howard (2007) note that symptoms of diseases can mimic organic brain disorders. They go on to explain that CI in a homeless person could indicate AIDS‐related dementia, prolonged depression, side effects from medication, or self‐medication with psychoactive substances. Ennis et al. (2015) also note that symptoms associated with mental health problems and substance abuse are common to post‐concussive syndrome. The findings from this review echo Burra et al.’s (2009) concerns that research in this field does not do enough to measure the impact of co‐occurring factors. As Backer and Howard explain, “For many homeless people, their impairments may come from more than one source, and the pattern of their cognitive problems may shift over time” (Backer & Howard, 2007, p377).

### 4.2 Experiences of homelessness

#### 4.2.1 Risks factors and perpetuators

Studies which reported risk factors to homelessness highlighted the social and economic instability experienced by persons with CI. In particular, persons with CI were likely to become homeless after a relationship breakdown and struggled with the bureaucracy of maintaining employment and housing. Given the overlap of symptoms, socioeconomic disadvantages and co‐occurring conditions experienced by persons with CI, it is unsurprising that risk factors to, and perpetuators of, homelessness were similar across all three categories. In some instances, however, these were emphasised according to the current understanding of impairment. For example, studies of ASD discussed social difficulties which result in barriers to housing echoing an autistic symptom of “persistent difficulties with social communication and interaction” (NAS, 2018a). Furthermore, the difficulties Pritchard's participants encountered substantiates anecdotal reports of autistic entrenched rough sleepers who are unwilling to change the comfort of routine for the unknown, despite the potential benefit to health and well‐being (Government W. W. A., 2011; Innovation & Good Practice Team, 2015). It was also the case that misunderstanding a particular CI could lead to or perpetuate homelessness. This was particularly common where a “higher functioning” individual was not identified as needing extra support, or where co‐occurring mental health issues were not identified or met with appropriate adaptation of service delivery. These findings echo concerns that awareness and understanding of CI amongst those working in the housing sector and homelessness services are not sufficient to meet the multitude of complex needs such individuals may present (Trueland, 2009).

Structural and personal life events such as losing one's job or the death of a carer can be understood as catalysts to homelessness. In this case, no single event causes homelessness, which is instead interpreted as the result of both personal circumstance and structural factors outside of one's control (Shelter, 2018). For people with CI, this review has shown how complex and interlinking factors caused by both impairment effects and the way CIs are received and supported can increase risk of homelessness. Therefore, to identify multiple risk factors, it is important to consider CI from a social‐relational understanding of disability. Such cumulative causes were recognised by a minority of authors in this review. Bymaster et al. (2017) discussed life experiences working in multifactorial ways alongside socioeconomic disadvantages to increase risk of homelessness and decrease possibility of rehabilitation. Similarly, Topolovec‐Vranic et al. (2013) recognised that sustaining a TBI could not be extracted from the context of other issues such as mental health problems or poverty. The findings of this review also support claims that, in some instances, the relationship between homelessness and acquired cognitive impairment may be bidirectional. In this case, factors associated with homelessness such as increased likelihood of assault, substance misuse, and mental health problems may lead to further acquired impairments such as sustaining a TBI or developing alcohol‐related brain damage (Backer & Howard, 2007; Barnes et al., 2015; Gilchrist & Morrison, 2005).

#### 4.2.2 Risks and perpetuators “same but amplified”

Risk factors to homelessness were heightened by the presence of CI. However, these factors were no different to those affecting the general population. UK homeless charity Crisis (2018) cites a number of social and economic reasons for becoming homeless. These broadly reflect the risk factors found by studies in this review and include relationship breakdown, release from prison, abuse, mental health issues, and loss of employment. In her 2015 study, Campbell notes that although risk factors are the same for persons with ASD as the general population, there are unique structural and individual factors which increase their severity for high‐functioning ASD adults. The findings of this scoping review widen this concern to include all individuals with CI. This can be exemplified by taking a common cause of homelessness, such as unemployment, and examining its relationship with the CI population. For example, 17% of adults with a learning disability are estimated to be in full time employment in the United Kingdom in comparison to 64% of the general population (ONS, 2016a, 2016b ).

### 4.3 Implications

#### 4.3.1 For future research

The majority of included studies were prevalence based and substantiated claims that all types of CI are overrepresented in the homeless population. Due to variety in definition and measures adopted, it is suggested that further prevalence studies will continue to produce differing percentages of CI rates in the homeless, which, nonetheless, show over‐representation in this population. Shifting focus to conceptualising CIs and examining how measures are affected by variables such as being homeless or having a co‐occurring mental health problems is necessary before types of CI can be more accurately recorded.

The usefulness of prevalence studies is questioned, however, as, unless they are acted upon, they do little to address the issue at hand. Studies which did not focus on prevalence tended to be concerned with the experiences of learning disability homeless, which is unsurprising given the social model of disability's permeation of this field. These studies were more effective at identifying risks factors to homelessness for these populations. In light of this, future research should focus on the experiences and perspectives of those with CI. The adoption of social or social‐relational understandings of disability across multiple disciplines could help facilitate this.

It is also of note that only two studies could be considered longitudinal. While it is difficult to follow up with homeless populations, research which attempts to examine the impact of CIs on homeless populations over time would be better positioned to identify barriers to rehabilitation. Types of homeless population should also be carefully considered. The fact that most studies recruited from shelters and the majority of participants were male reduces generalisability. In line with current trends in disability research, it is lastly suggested that more is done to include those with CI in the research about them. When conducted well, inclusive research has the potential for unique contribution and actualisation of social change (Walmsley, Strnadova, & Johnson, 2017). Including cognitively impaired persons in the research process could draw attention to important ethical issues, such as gaining informed consent, which were not explored sufficiently in studies.

#### 4.3.2 For service delivery and policy

The multifactorial risk factors identified suggest that persons with CI need to be supported by services which take a whole person approach. The high rate of co‐occurrence between CIs and other disorders suggests the need for integrated interventions to tackle issues concurrently (Baker & Howard, 2007). In order to do so, CIs and co‐occurring conditions need to be identifiable. This requires an increased awareness amongst practitioners accompanied by adaptations to service delivery to maximise accessibility. Such adaptations might include making easy read versions of information or supplying advocates to be present at welfare assessments. Research also suggests that the treatment of the complex and interlinking problems associated with homelessness should occur alongside, as opposed to prior to, rehousing efforts (Cornes, Manthorpe, Joly, & O'Halloran, 2014). Specific considerations must be made when sourcing suitable accommodation for cognitively impaired, homeless people. This review suggests people with CI may struggle socially, making it difficult for individuals to live in certain environments. Effort must be made to consider the suitability of setting before the placement decisions are made.

Recent changes to the UK's welfare system have come under criticism for their negative impact on persons with CIs (Headway, 2017; Homeless Link, 2013; NAS, 2017). Further concerns raised by the National Audit Office (2017) highlight an increase in local authority spending on homelessness management, at the expense of utilising funds to prevent it. Spending on the Supporting People program, designed to help vulnerable persons, such as those with complex mental health issues or LDs, live independently, has decreased by 59% since 2010. (National Audit Office, 2017; Welsh Government, 2017). Such austerity measures are inherently disadvantaging to persons with CIs. Policy makers must thus strive to challenge the barriers which restrict opportunity and quality of life. This means looking at the socioeconomic disadvantages experienced by persons with CI as well as the individual events which trigger homelessness. This could include redressing some of the welfare concerns raised here, or examining the relationship between CI and imprisonment, or the risk persons with CI are at when ageing carers pass away and no plan has been made for continued support. Until the stigmatisations and economic realities which continue to jeopardise their life outcomes are challenged, persons with CI will continue to be over‐represented in homeless populations.

## 5 LIMITATIONS

While this review focussed on homeless populations, some studies have been included which sampled from prisons, mental health services, and hospitals. These studies reported that people with CI experienced increased instances of homelessness and were picked up in database searches due to their use of “homeless*” in keywords, titles, or abstracts. It is acknowledged that their inclusion reduces the comparability of studies, but to exclude them would have limited access to information on the link between homelessness and CIs, particularly concerning developmental disabilities.

Despite the international scope of this review, some findings have been generalised to the UK policy context. It is acknowledged that differing social and economic climates may affect the extent to which CI is understood and supported internationally. The impact of this is reduced somewhat given the concentration of research in Canada, the United States, and the United Kingdom, and research which suggests the principle causes of homelessness are similar across Westernised countries (Crane et al., 2005) It is up to the reader to consider the relevance of research findings across globalised settings.

## CONFLICTS OF INTEREST

None known.

## ENDNOTE

1 ASD: autism spectrum disorder, TBI: traumatic brain injury, ABI: acquired brain injury, NDD: neurodevelopmental disability, ADHD: attention deficit hyperactivity disorder, william: Williams syndrome.
